# Piperlongumine selectively kills hepatocellular carcinoma cells and preferentially inhibits their invasion via ROS-ER-MAPKs-CHOP

**DOI:** 10.18632/oncotarget.3444

**Published:** 2015-01-31

**Authors:** Yong Chen, Ju Mei Liu, Xin Xin Xiong, Xin Yao Qiu, Feng Pan, Di Liu, Shu Jue Lan, Si Jin, Shang Bin Yu, Xiao Qian Chen

**Affiliations:** ^1^ Department of Pathophysiology, School of Basic Medicine, Tongji Medical College, Institute of Brain Research, Key Laboratory of Neurological Diseases, Ministry of Education, Hubei Provincial Key Laboratory of Neurological Diseases, Huazhong University of Science and Technology, Wuhan, China; ^2^ Department of Urology, Union Hospital, Huazhong University of Science and Technology, Wuhan, China; ^3^ Institute of Biochemistry and Cell Biology, Shanghai Institutes for Biological Sciences, Chinese Academy of Sciences, Shanghai, China; ^4^ Department of Pharmacology, School of Basic Medicine, Huazhong University of Science and Technology, Wuhan, China

**Keywords:** piperlongumine, hepatocellular carcinoma, oxidative stress, signaling pathway, chemotherapy

## Abstract

Hepatocellular carcinomas (HCC) are highly malignant and aggressive tumors lack of effective therapeutic drugs. Piperlongumine (PL), a natural product isolated from longer pepper plants, is recently identified as a potent cytotoxic compound highly selective to cancer cells. Here, we reported that PL specifically suppressed HCC cell migration/invasion via endoplasmic reticulum (ER)-MAPKs-CHOP signaling pathway. PL selectively killed HCC cells but not normal hepatocytes with an IC_50_ of 10-20 μM while PL at much lower concentrations only suppressed HCC cell migration/invasion. PL selectively elevated reactive oxygen species (ROS) in HCC cells, which activated or up-regulated downstream PERK/Ire 1α/Grp78, p38/JNK/Erk and CHOP subsequently. Administration of antioxidants completely abolished PL's effects on cell death and migration/invasion. However, pharmacological inhibition of ER stress-responses or MAPKs signaling pathways with corresponding specific inhibitors only reversed PL's effect on cell migration/invasion but not on cell death. Consistently, knocking-down of CHOP by RNA interference only reversed PL-suppressed HCC cell migration. Finally, PL significantly suppressed HCC development and activated the ER-MAPKs-CHOP signaling pathway in HCC xenografts *in vivo*. Taken together, PL selectively killed HCC cells and preferentially inhibited HCC cell migration/invasion via ROS-ER-MAPKs-CHOP axis, suggesting a novel therapeutic strategy for the highly malignant and aggressive HCC clinically.

## INTRODUCTION

Liver cancers are highly malignant and aggressive tumors, making it the third leading cause of cancer death in the world [1]. Hepatocellular carcinoma (HCC) is the most common type of liver malignant tumors in adults. HCC usually occurs following a viral hepatitis infection (hepatitis B or C) or cirrhosis, in which complicated pathological processes induced by reactive oxygen species (ROS) are heavily involved [2]. ROS is considered as a two-edge sword in tumorigenesis and development. At the initial stage in HCC development, ROS or oxidative stress-responses mainly stimulate hepatocarcinogenesis or promote HCC development by activating pro-oncogenic gene activities or oncogenic signaling pathways such as mitogen-activated protein kinases (MAPKs) [3, 4]. Along with the progression of HCC, ROS overproduction or long-lasting oxidative stress-responses would tend to induce cell death or prevent cancer development in HCC [1, 5]. Therefore, modulating homeostasis of ROS or oxidative stress-responses has become an important therapeutic strategy for HCC [5, 6]. Accumulative evidences support that oxidants have therapeutic effects in cancer treatment and the underlying mechanisms have been well studied [5, 7-11]. It is well-known that ROS may alter various cellular signaling pathways profoundly depending on different cellular contexts and MAPKs (i.e., p38, JNK and Erk) are canonical ROS-responsive signaling pathways [8-12]. Until now, the exact mechanisms of oxidative stress-responses in HCC development and treatment remain far from clear.

Piperlongumine (PL) is a biological active alkaloid existing largely in the long pepper (*Piper longum* L). PL has been traditionally used for treating gastrointestinal and respiratory diseases in Ayurvedic medicine [12]. Recently, PL was identified as a highly reliable and potent cytotoxic compound in killing cancer cells in screening study [13]. PL selectively kills cancer cells but leave normal cell intact as PL induces ROS accumulation only in cancer cells [8, 9, 13]. The PL induced selective accumulation of ROS in cancer cells represents a novel therapeutic strategy for cancers [8, 9, 13, 14]. It is reported that PL might exert its cytotoxicity by activating p38 [9,11], JNK [9], Erk [15], Akt [16, 17], promoting protein glutathionylation [18], or suppressing NFκB activities [19] in different types of cancer cells. Further exploring the anticancer effects as well as its underlying mechanisms of PL is required for its clinical applications.

Endoplasmic reticulum (ER), a specific organelle for Ca^2+^ storage and proper protein folding/maturation, plays an important role in regulating ROS homeostasis and stress-responses [20]. Upon various pathological stimuli such as ROS or misfolded/unfolded proteins accumulation, ER homeostasis is disturbed and ER stress-responses are induced, leading to the activation of various downstream signaling pathways such as MAPKs and the induction of C/EBP homologous protein (CHOP) [21, 22]. Consequently, stressed cells may either restore its homeostasis or undergo programmed cell death such as apoptosis or autophage [23]. In various cancer cells including HCC cells, enhanced ER stress-responses have been well documented [24-26]. However, the effects of ER stress-responses (either promoting or inhibiting cancer development) vary depending on specific ER-downstream signaling pathways in specific cellular contexts [24, 27]. Considering the central role of ER in oxidative stress-responses in HCC, it is likely that ER-mediated stress-responses and its downstream signaling pathways might be heavily involved in PL's biological effects in HCC cells.

In the present study, we examined the anticancer effects of PL on HCC cells *in vitro* and *in vivo*, and further investigated the underlying mechanisms. Our data demonstrated that PL preferentially suppressed HCC cell migration and invasion via ER-MAPKs-CHOP depending on ROS.

## RESULTS

### Piperlongumine selectively kills HCC cells but not normal hepatocytes

**Figure 1:**
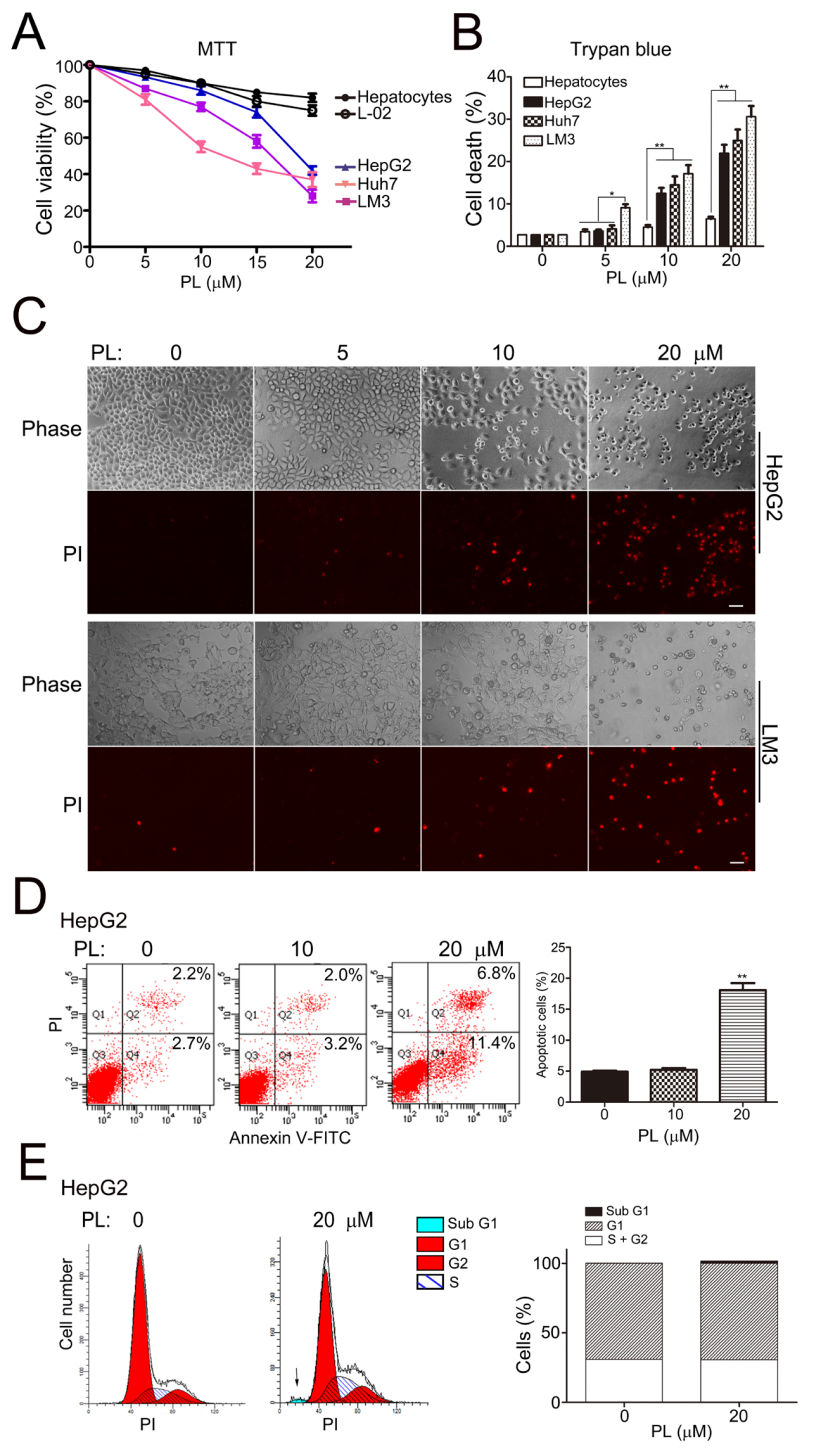
Piperlongumine selectively killed HCC cells but not normal hepatocytes (A) Results of MTT assays showed the selective cytotoxicity of PL in HCC cells. HCC cell lines (HepG2, Huh7 and LM3), normal hepatic cell line (L-02) and primary rat hepatocytes were grown in 96-well plates for 24 h and subjected to PL treatment (0, 5, 10, 15 or 20 μM) for 24 h. Cell viability was measured by MTT assays (n=3). All values represented mean ± SEM of three independent experiments. (B) Statistical analysis of cell death in PL-treated HCC cells or primary hepatocytes as measured by trypan blue exclusion assays. *P<0.05, **P<0.01 (n=3). (C) Representative micrographs of PI staining showed cell death in PL-treated HepG2 and LM3 cells. Bar, 20 μm. (D) Representative results of FCM analysis showed apoptosis in PL-treated HepG2 cell. HepG2 cells were treated with PL (0, 10 or 20 μM) for 24 h. After annexin V-PI double staining, cells were subjected to FCM analysis. The percentages of cells in the Q2 together with Q4 quarters were calculated as apoptotic cells for statistical analysis in the right panel. **P<0.01 *vs.* PL 0 μM (n=3). (E) Representative results of FCM analysis showed the effects of PL in cell cycle of HepG2 cells. HepG2 cells were treated with 20 μM PL for 24 h. After PI staining, cells were subjected for FCM analysis. The arrow indicated the sub-G1 population.

We tested the cytotoxic effects of PL in human HCC cell lines to evaluate the therapeutic potential of PL for HCC. MTT assays demonstrated that 24 h of PL treatment induced a significant reduction in the cell viability of various HCC cells (HepG2, Huh7, LM3) in a concentration-dependent manner (PL 0, 5, 10, 15 and 20 μM), but had little effect in that of primary rat hepatocytes and normal hepatic L-02 cells (Fig. 1A). The half maximal inhibitory concentration (IC_50_) of PL in HCC cells was in the range of 10-20 μΜ. Trypan blue assay showed that the rate of cell death was significantly increased in HepG2, Huh7 and LM3 cells at 24 h of PL treatment in a dose-dependent manner (5-20 μM) (Fig. 1B). Consistently, the rate of cell death as revealed by propidium iodide (PI) staining (PI-positive nuclei) was evidently increased in HepG2, LM3 (Fig. 1C) and Huh7 (Supplemental Figure 1) cells at 24 h of PL treatment in a dose-dependent manner (5-20 μM). Further, the number of apoptotic cells as revealed by annexin V staining (annexin V-positive cells) was significantly increased at 24 h of PL-treatment (20 μM) in HepG2 cells (Fig. 1D). In addition, flow cytometry (FCM) analysis showed that at 24 h of PL-treatment (20 μM), the percent of sub-G1 cells (representing apoptotic cells) was increased in HepG2 cells while the ratio of G1/(S+G2) was not altered (Fig. 1E). Taken together, PL selectively and effectively induced cell death including apoptosis in HCC cells.

### Piperlongumine preferentially suppresses HCC cell migration and invasion *in vitro*

**Figure 2:**
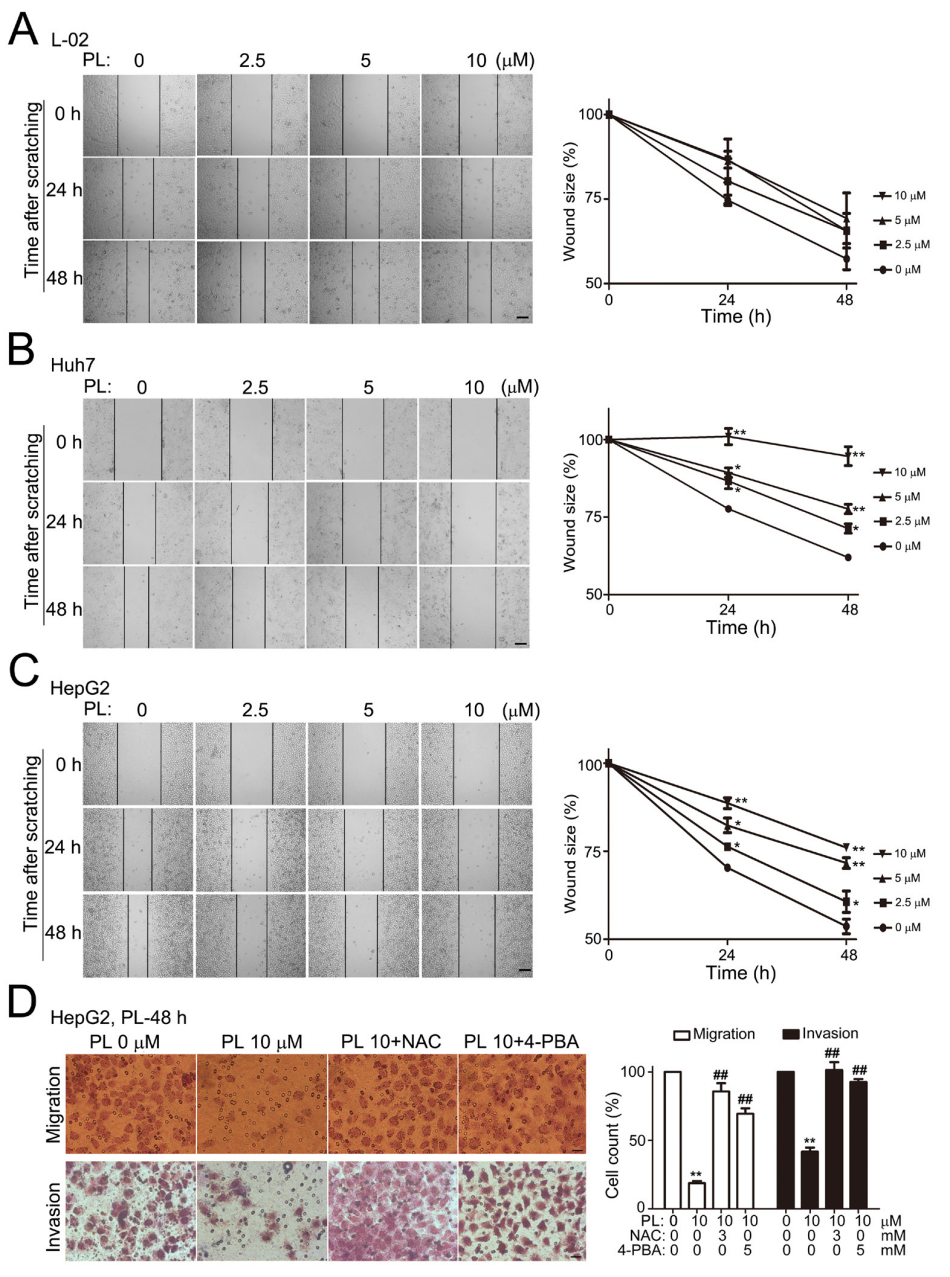
Piperlongumine selectively suppressed HCC cell migration or invasion *in vitro* (A) Representative micrographs showed the effects of PL on L-02 cell migration after scratching. Confluent L-02 cells (normal hepatic cell line) in 35-mm culture dishes were subject to cell scratching with yellow pipettes. PL was added to the cultures with fresh culture media after brief culture rinsing. The scratch-wounds were photographed at various time points (0, 24 and 48 h) after cell scratching and the wound sizes were measured. Bar, 100 μm. Statistical analyses (right panel) demonstrated that PL with 10 μM did not alter L-02 cell migration significantly (n=3). (B-C) Representative micrographs showed the effects of PL on Huh7 (B) and HepG2 (C) cell migration after cell scratching. Bar, 100 μm. Statistical analyses (right panel) demonstrated that PL with 2.5-10 μM significantly altered Huh7 cell migration 48 h after cell scratching (n=3). *P<0.05, **P<0.01 *vs.* corresponding PL 0 μM control (n=3). (D) Representative micrographs showed the effects of PL on HepG2 cell migration and invasion. HepG2 cells were seeded into the upper chamber of transwell apparatus without (upper panel) or with (lower panels) matrigel. Drugs (PL alone or together with NAC or 4-PBA) were added to the culture 24 h after cell seeding. Cell migration (upper panels) and invasion (lower panels) were induced by FBS-containing media in the lower chamber. Migrated and invaded cells in the lower surface of the filters were stained and microphotographed 24 h after serum induction. Bar, 20 μm. Statistical analyses (right panel) demonstrated migrated or invaded HepG2 cells were significantly reduced upon PL treatment while co-treatment of NAC or 4-PBA significantly reversed the effects of PL on cell migration or invasion. ^**^P<0.01 vs PL-0 μM and ^##^*P*<0.01 vs corresponding PL 10 μM control (n=3).

HCC are highly malignant tumors and cancer cell invasion or metastasis is the major cause of death. We examined the effects of PL on the ability of HCC cell migration and invasion *in vitro*. In the scratch-wound healing assay, cells along the wound-edge would migrate into the nude space after scratching and the scratch-wound would heal continuously (represented by the reduced scratch-wound sizes between the opposite scratching lines). In normal hepatic L-02 cells, PL treatment (within 10 μM) had no evident effect on the migration and the wound-healing speed was not altered within 48 h of PL treatment (Fig. 2A). In Huh7 (Fig. 2B) and HepG2 (Fig. 2C), however, PL prominently inhibited HCC cell migration and reduced wound-healing speed at 24 or 48 h of PL treatment in a dosage-dependent manner within 2.5-10 μM. Statistical analysis demonstrated that PL at lower concentrations (2.5, 5 or 10 μM) significantly reduced wound sizes of Huh7 (Fig. 2B) or HepG2 (Fig. 2C) cells while cell death in HepG2 or Huh7 cells was not altered at 48 h of PL treatment at 10 μM (Supplemental Fig. 2). Consistently, PL (10 μM) prominently reduced HepG2 cell migration at 48 h of PL treatment in the transwell migration assays (left upper panels, Fig. 2D). Further, PL (10 μM) significantly reduced invaded HepG2 cells in the transwell invasion assay at 48 h after PL-treatment (left lower panels, Fig. 2D). Statistical analysis demonstrated that migrated or invaded HepG2 cells were significantly reduced at 48 h of PL treatment (10 μM) (right panel, Fig. 2D). These evidences together demonstrated that PL preferentially suppressed HCC cell migration/invasion at lower PL concentrations.

### Piperlongumine induces ROS accumulation to exert its anti-cancer effects on HCC cells

**Figure 3:**
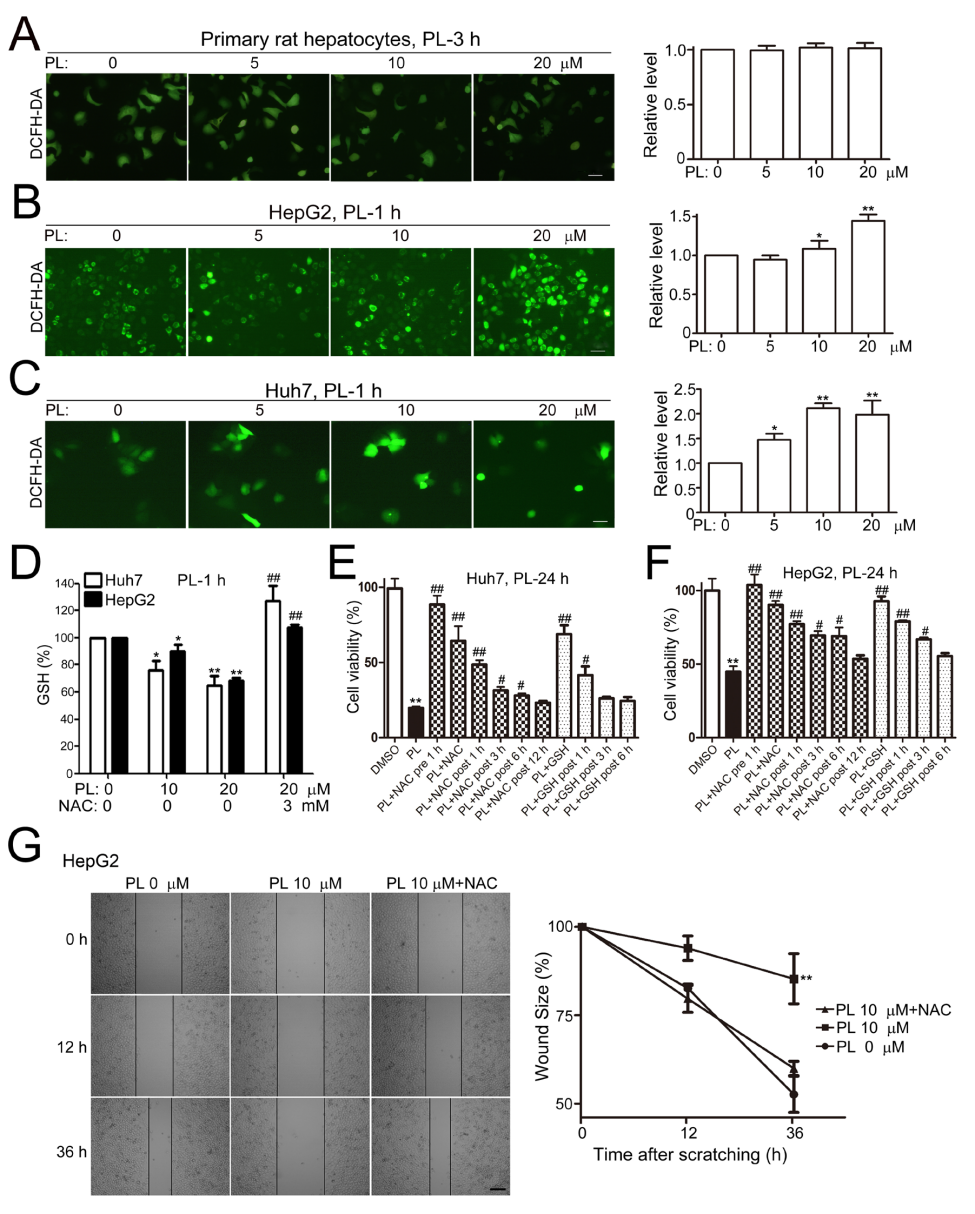
Piperlongumine induced ROS accumulation to kill and suppress HCC cell migration (A) Effects of PL on the ROS level in primary rat hepatocytes. Cultured primary rat hepatocytes were treated with PL and stained with DCFH-DA. Bar, 20 μm. Representative micrographs showed that the fluorescent intensity of DCFH-DA was not altered within 3 h after PL treatment. For statistical analysis, the mean DCFH-DA fluorescent intensity (representing cellular ROS level) was measured from 9 random fields for each culture. All values represented mean ± SEM of three independent experiments. (B-C) Effects of PL on the ROS level in HepG2 (B) and Huh7 (C) cells. Bar, 20 μm. *P<0.05 and **P<0.01 *vs.* corresponding PL 0 μM (n=3). (D) Effects of PL on the GSH level in HCC cells. NAC was pretreated for 1 h and then co-treated with PL for another 1 h. *P<0.05, **P<0.01 *vs.* PL 0 μM control. ^##^*P*<0.01 *vs.* corresponding PL 20 μM control. (E-F) Effects of antioxidants on PL-induced cytotoxicity in Huh7 (E) and HepG2 (F) cells. NAC (3 mM) or GSH (5 mM) was administrated either before PL (20 μM) administration (PL+NAC pre), simultaneously with PL (PL+NAC/GSH) or after PL treatment (PL+ NAC/GSH post). Cell viability was measured by MTT assays. **P<0.01 *vs.* DMSO control; ^#^*P*<0.05 or ^##^*P*<0.01 *vs.* PL 20 μM control (n=3). (G) Effects of NAC on PL-suppressed HepG2 cell migration after cell scratching. NAC (3mM) was administrated simultaneously with PL after cell scratching. Bar, 100 μm. ^**^P<0.01 *vs.* PL 0 μM or PL 10 μM+NAC (n=3).

We then examined whether ROS accumulation is required for PL's anti-cancer effects in HCC cells as previous studies have shown that ROS is the major downstream player of PL's action in many types of cancer cells [13]. The fluorescent intensity of 2′-,7′-Dichlorofluorescin diacetate (DCFH-DA, a specific ROS indicator) in primary hepatocytes (Fig. 3A) or L-02 cells (Supplemental Fig. 3A) was not altered at 3 h of PL treatment (5, 10 and 20 μM). In HepG2 (Fig. 3B), Huh7 (Fig. 3C) and LM3 cells (Supplemental Fig. 3B), however, the fluorescent intensities of DCFH-DA were evidently enhanced at 1 or 3 h of PL treatment. Statistical analyses demonstrated that the intracellular ROS levels were significantly increased in HepG2 and Huh7 cells at 1 h of PL treatment (Fig. 3B and 3C). Pre-incubation of antioxidant N-acetyl-L-cysteine (NAC, 3 mM) completely abolished the effect of PL on ROS induction at 3 h of PL treatment in LM3 (Supplemental Fig. 3B) or HepG2 cells (Supplemental Fig. 3C). Consistently, intracellular glutathione (GSH) levels were significantly reduced in HepG2 or Huh7 cells at 1 h of PL treatment (Fig. 3D). Pre-treatment of NAC (3 mM) reversed PL's effect on GSH reduction (Fig. 3D). Pre-treatment of NAC or GSH completely reversed PL-induced cell death in Huh7 cells (Fig. 3E) and HepG2 cells (Fig. 3F) while delayed administration of antioxidants partially reversed PL-induced cell death in HCC cells at a time-dependent manner (Fig. 3E and 3F). In addition, administration of NAC completely reversed PL-suppressed cell migration in HepG2 cells after cell scratching (Fig. 3G). Consistently, NAC abolished the PL's suppression effect on cell migration in the transwell migration assay (left upper panels, Fig. 2D) and cell invasion in the transwell invasion assay (left lower panels, Fig. 2D).

### Piperlongumine induces ER stress-responses which preferentially suppresses HCC cell migration/invasion

**Figure 4:**
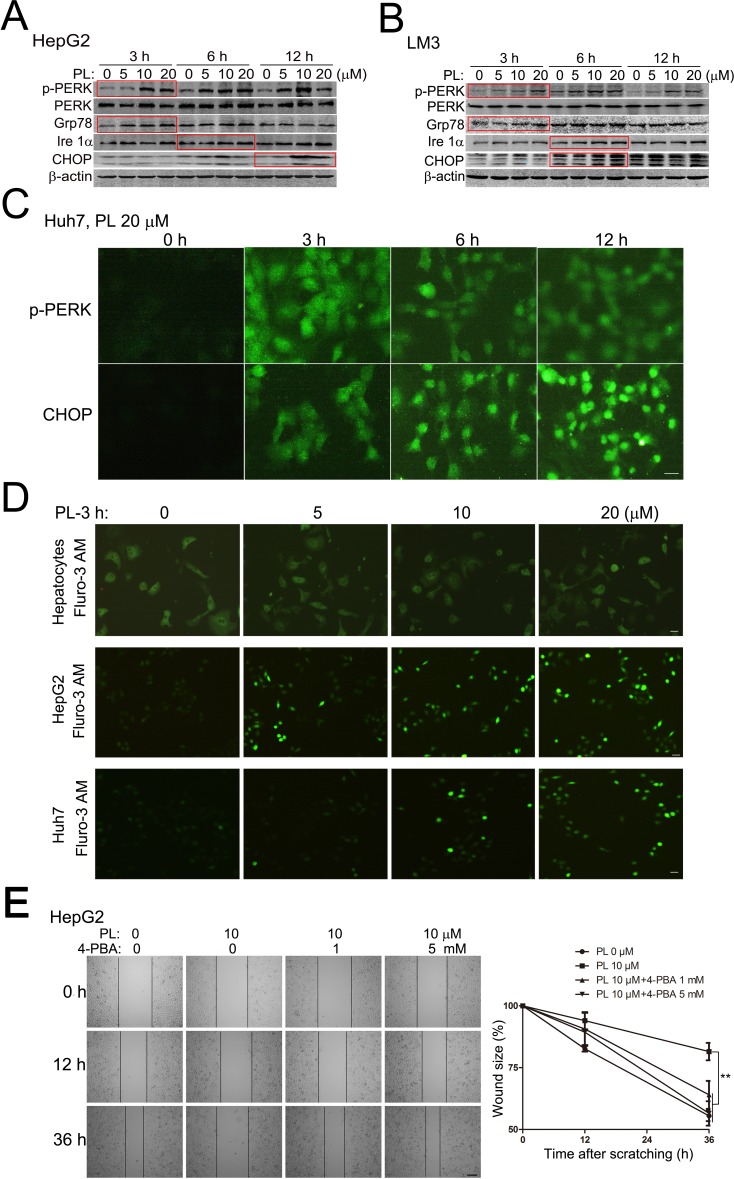
Piperlongumine induced ER stress-responses to suppress HCC cell migration preferentially (A-B) Representative results of Western blotting analysis of specific ER stress-response proteins in PL-treated HepG2 (A) and LM3 (B) cells. HepG2 or LM3 cells were treated with 0, 5, 10 and 20 μM of PL for 3, 6 or 12 h. Equal amounts of total proteins were subjected to Western blotting analysis with designated antibodies. The red box indicated the most evident changes of p-PERK, Grp78, Ire 1α or CHOP in PL-treated HepG2 (A) and LM3 (B) cells. (C) Representative micrographs of fluorescent immunostaining showed PL (20 μM) activated p-PERK and up-regulated CHOP in Huh7 cells. Bar, 20 μm. (D) Representative fluorescent micrographs of Fluro-3AM showed that PL enhanced free calcium concentration in PL-treated HepG2 or Huh7 cells but not primary rat hepatocytes. Bar, 20 μm. (E) Effects of ER stress-response inhibitor 4-PBA on PL-suppressed HepG2 cell migration after cell scratching. Bar, 100 μm. ^**^P<0.01 (n=3).

The downstream signaling pathways of ROS which is responsible for PL's anticancer effects remain elusive. We examined the role of ER stress-responses in PL-treated HCC cells as the ROS-ER axis plays a pivotal role in controlling ROS-mediated homeostasis and cellular effects [20]. Western blotting analysis showed that PKR-like ER kinase (PERK, a specific ER-stress responsive protein) was prominently activated in HepG2 (Fig. 4A) and LM3 (Fig. 4B) cells at various time points (e.g., 3 h as indicated by the red rectangular box) of PL treatment in a dosage-depedent manner (0, 5, 10 and 20 μM). Fluorescent cytoimmunostaining assay also clearly showed that the level of phosphorylated PERK (p-PERK) was evidently increased in Huh7 (Fig. 4C) and HepG2 cells (Supplemental Fig. 4A) at various time points (3, 6 and 12 h) of PL treatment (20 μM). In addition, other ER-endocking proteins such as glucose-regulated protein 78 (Grp78, i.e., BiP) and inositol-requiring enzyme-1α (Ire 1α) were also clearly up-regulated at various time points of PL treatment in HepG2 (Fig. 4A) and LM3 (Fig. 4B) cells. Consistently, the downstream product of ER stress-response, CHOP, was also up-regulated evidently at various time points of PL treatment in HepG2 (Fig. 4A and Supplemental Fig. 4A), LM3 (Fig. 4B) and Huh7 cells (Fig. 4C). ER is a major organelle storing Ca^2+^ which is released into cytosol to activate downstream signaling pathways upon various stimuli including ROS [28]. In primary rat hepatocytes, the concentration of cytoplasmic free Ca^2+^ as measured by Fluro-3 AM (a specific fluorescent probe for free calcium) was not altered at 3 h of PL treatment (0, 5, 10 and 20 μM) (upper panels, Fig. 4D). In HepG2 (middle panels, Fig. 4D) and Huh7 (lower panels, Fig. 4D) cells, however, the concentration of cytoplasmic free Ca^2+^ was prominently increased at 3 h of PL treatment in a dose-dependent manner (0-20 μM). These evidences together verified that PL induced ER stress-responses in HCC cells.

We then examined the roles of ER stress-responses in PL's anticancer effects in HCC cells by using specific ER stress-response inhibitor 4-phenylbutyric acid (4-PBA) [29, 30]. MTT assay revealed that 4-PBA did not alter PL-induced cell viability reduction in HepG2 or LM3 cells (Supplemental Fig. 5A and 5C). Consistently, results of PI staining showed that 4-PBA did not alter PL-induced cell death in HepG2 cells (Supplemental Fig. 5B). However, 4-PBA completely abolished PL-suppressed cell migration in HepG2 (Fig. 4E) and Huh7 cells (Supplemental Fig. 6) at various time points after scratching. Consistently, 4-PBA significantly reversed PL-suppressed HepG2 cell migration in transwell migration assays and HepG2 cell invasion in the transwell invasion assays (Fig. 2D). The reversing effects of 4-PBA on PL's effects on HCC cell migration were close to those of NAC, suggesting that PL inhibited HCC cell migration or invasion via ROS-ER-mediated signaling preferentially.

### Piperlongumine activates MAPKs signaling pathways which preferentially suppress HCC migration

**Figure 5:**
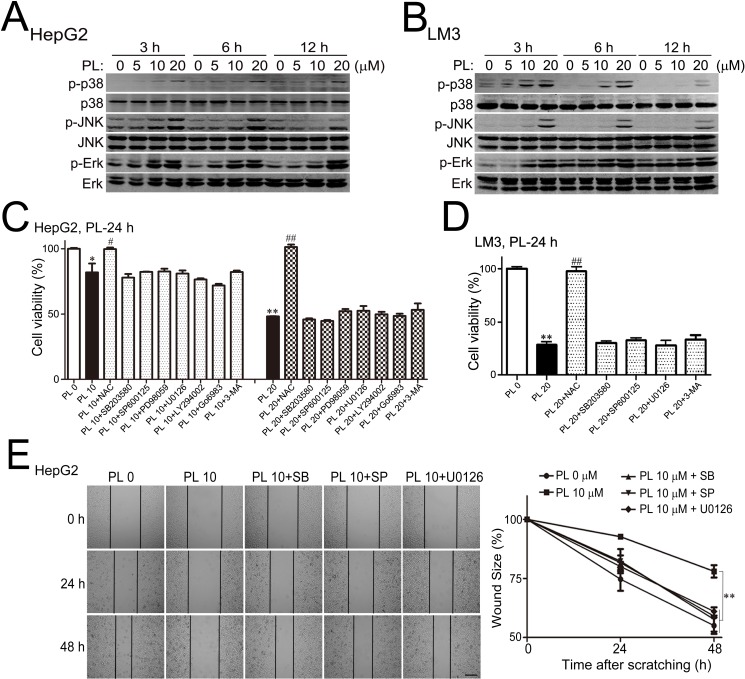
Piperlongumine activated MAPKs to suppress HCC cell migration preferentially (A-B) Representative results of Western blotting analysis demonstrated that p38, JNK and Erk were prominently activated in PL-treated HepG2 (A) and LM3 (B) cells in a dosage-dependent manner. (C-D) Effects of specific inhibitors on the cell viability in PL-treated HepG2 (C) and LM3 (D) cells as measured by MTT assays. PL were administrated alone or together with other drugs (i.e., SB203580 10 μM, SP600125 10 μM, PD98059 10 μM, LY294002 10 μM, U0126 10 μM, Go6983 10 μM or 3-MA 10 μM). *P<0.05, **P<0.01 *vs.* PL 0; ^#^*P*<0.05, ^##^*P*<0.01 *vs.* corresponding PL 20 (n=3). (E) Effects of specific MAPKs inhibitors on PL-suppressed HepG2 cell migration after cell scratching. PL (10 μM) was administrated together with 10 μM of SB203580, SP600125 or U0126. Bar, 100 μm. ^**^P<0.01 (n=3).

MAPKs are canonical ROS- and ER-stress responsive signaling pathways that control cell death under oxidative stress [31, 32]. Results of Western blotting analysis showed prominent activation of p38, JNK and Erk in HepG2 (Fig. 4A) and LM3 (Fig. 4B) cells at various time points of PL treatment in a dose-dependent manner (0, 5, 10 or 20 μM). We then examined whether the activation of MAPKs contributed to PL's anti-cancer effects in HCC cells. Pharmacological inhibition of p38, JNK or Erk signaling pathway by using corresponding inhibitor SB203580, SP600125 or U0126 did not alter PL's effects on cell death in HepG2 (Fig. 5C) or LM3 cells (Fig. 5D). However, suppressing p38 (SB203580), JNK (SP600125) or Erk (U0126) signaling pathway prominently reversed PL's effects on HepG2 cell migration at 24 or 48 h after cell scratching (left panels, Fig. 5E). Statistical analysis demonstrated that the wound sizes were significantly increased in SB203580/SP600125/U0126 plus PL 10 μM treatment groups as compared to that in PL 10 μM treatment group in HepG2 cells (right panel, Fig. 5E). These evidences suggested that the activation of MAPKs mainly contributed to PL's effect on cell migration.

### Piperlongumine functions through ROS-ER-MAPKs-CHOP axis in HCC cells

**Figure 6:**
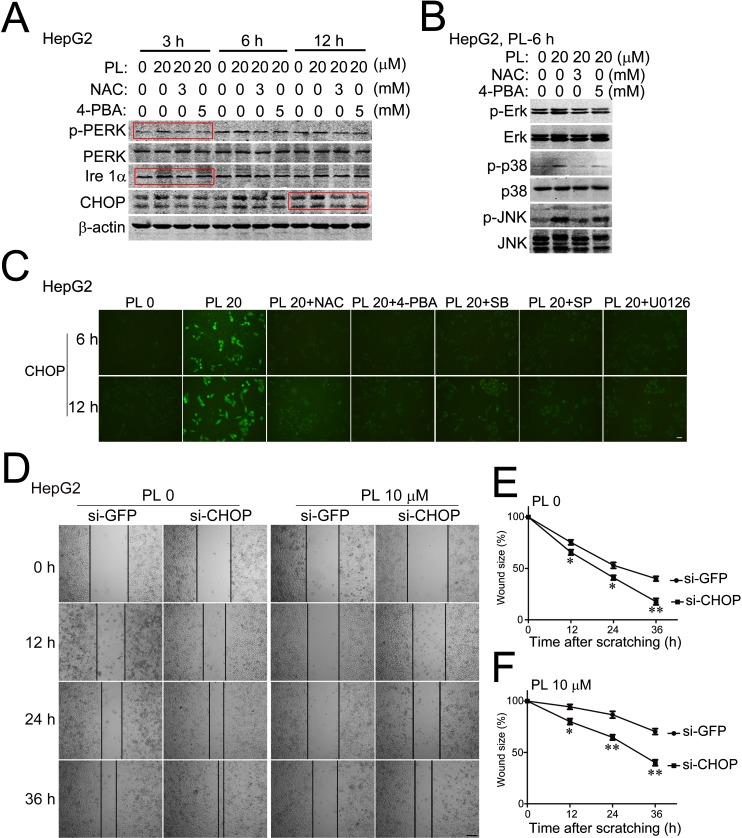
Piperlongumine suppressed HCC cell migration via ROS-ER-MAPKs-CHOP signaling pathway (A) Representative results of Western blotting analysis showed the effects of NAC or 4-PBA on the expression of specific ER stress-response proteins (p-PERK/PERK, Ire 1α and CHOP) in PL-treated HepG2 cells. The red boxes indicated the most evident reversal effect of NAC or 4-PBA. (B) Representative results of Western blotting analysis showed the effects of NAC or 4-PBA on the activation of p38/JNK/Erk. (C) Representative micrographs of fluorescent cytoimmunostaining showed the effects of NAC, 4-PBA or MAPKS inhibitors on CHOP expression in PL-treated HepG2 cells. Bar, 20 μm. (D) Effects of knocking-down CHOP by RNA interference on HepG2 cell migration. HepG2 cells were transfected with small interfering RNA specific for CHOP (si-CHOP) for 2 days and then subjected to cell scratching and PL treatment. Small interfering RNA specific for green fluorescent protein (si-GFP) was used as non-specific control. Bar, 100 μm. (E) and (F) Statistical analyses demonstrated that silencing CHOP (si-CHOP) significantly accelerated the speed of wound-healing of HepG2 cells after scratching without (E) or with PL treatment (F). *P<0.05, **P<0.01 *vs.* corresponding si-GFP (n=3).

We then verified the existence of ROS-ER-MAPKs-CHOP axis in PL-treated HCC cells by using the corresponding inhibitors. Results of Western blotting analysis showed that co-incubation of antioxidant NAC with PL completely abolished PL-activated PERK (Fig. 6A, indicated by the rectangular box) and p38/JNK/Erk (Fig. 6B), and PL-up-regulated Ire 1α and CHOP (Fig. 6A) in HepG2 cells at various time points of PL treatment. The ER stress inhibitor 4-PBA showed similar inhibitory effects on ER-MAPKs-CHOP signaling pathways compared to NAC (Fig. 6A and 6B). Consistently, results of fluorescent cytoimmunostaining assay also showed that NAC and 4-PBA evidently reduced the elevated level of p-PERK (Supplemental Fig. 4B) or CHOP by PL (Fig. 6C) at various time points of PL treatment in HepG2 cells. Further, suppressing p38/JNK/Erk signaling pathways by SB203580/SP600125/U0126 correspondingly evidently reduced PL-elevated CHOP at 6 or 12 h of PL treatment in HepG2 cells (Fig. 6C). These evidences demonstrated that PL activated ER-MAPKs-CHOP axis signaling pathways via ROS-dependent mechanisms.

Finally, we examined the role of CHOP in HCC cell migration by knocking-down CHOP via small RNA interference technique. Transfection of small interfering RNA specific to CHOP (si-CHOP) [30] effectively reduced the expression of endogenous CHOP in HepG2 cells (Supplemental Fig. 7A) but did not affect the viability of HepG2 cells (MTT assay) at 24 h of PL treatment (Supplemental Fig. 7B). However, transfection of si-CHOP evidently accelerated HepG2 cell migration at various time points (12, 24 and 36 h) after cell scratching as compared to corresponding si-GFP (green fluorescent protein) controls (Fig. 6D). Statistical analysis demonstrated that knocking-down of CHOP (si-CHOP) significantly reduced the wound-size of HepG2 culture at various time points after scratching without or with PL treatment (PL 0, Fig. 6E and PL 10 μM, Fig. 6F) as compared to corresponding si-GFP controls. These results supported that CHOP was required for suppressing HCC cell migration and was a critical downstream target of PL's action in HCC cell migration.

### Piperlongumine activates ER-MAPKs-CHOP axis signaling and reduces HCC *in vivo*

**Figure 7:**
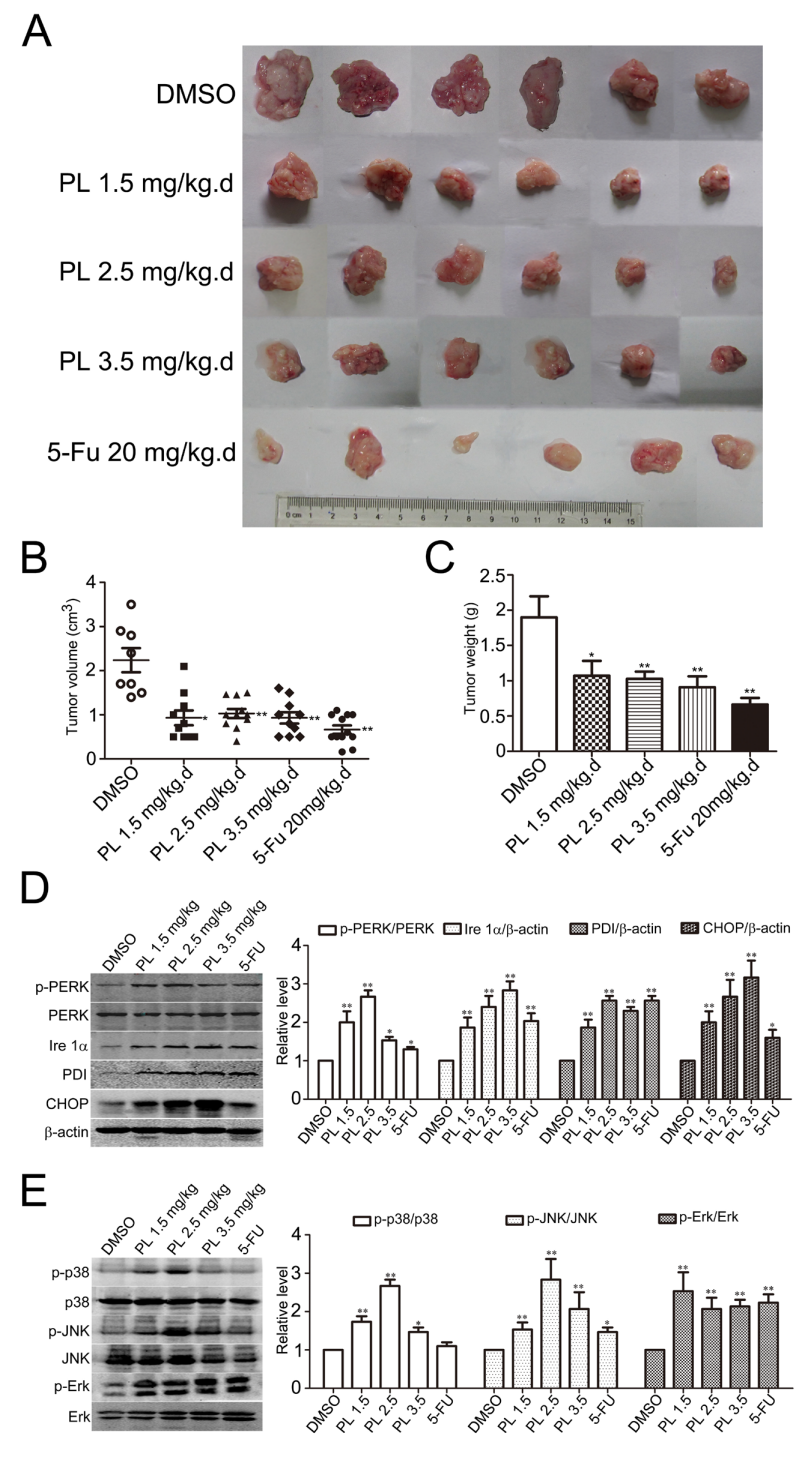
Piperlongumine suppressed HCC development and activated ER-MAPKs-CHOP signaling pathway (A) Representative inoculated HCC tumors from mice treated with PL. Equal amounts of H22 cells were subcutaneously injected into the left flank of adult mice. PL, 5-Fu or DMSO was administrated for 14 days (once per day) and then the tumor xenografts were removed completely from tissues. (B-C) Statistical analyses demonstrated that the average volume (B) and weight (C) of HCC xenografts from PL-treated mice were significantly reduced. *P<0.05, **P<0.01 *vs.* DMSO group (n=8). (D) Representative Western blotting analysis and statistical analysis of p-PERK/PERK, Ire 1α, PDI and CHOP from xenografts. *P<0.05, **P<0.01 *vs.* corresponding DMSO control (n=5). (E) Representative Western blotting analysis and statistical analysis of p-p38/p38, p-JNK/JNK and p-Erk/Erk from xenografts. *P<0.05, **P<0.01 *vs.* corresponding DMSO control (n=5).

After clarifying the anticancer effects and mechanisms of PL in HCC cells *in vitro*, we examined the role of PL in HCC-bearing mice. Equal amount of murine ascetic H22 hepatoma cells was subcutaneously inoculated into the flank side of adult male Kunming mice [33-35]. After 24 h of inoculation, PL was administrated once a day by intraperitoneal (i.p.) injection for 14 days consecutively and then tumor xenografts were isolated. (Fig. 7A) showed that the tumor xenografts from PL-treated groups were evidently smaller than those from DMSO control group. Statistical analysis demonstrated that the average volume (Fig. 7B) and weight (Fig. 7C) of the tumor xenografts from PL treatment groups were both significantly decreased as compared to that from DMSO control group. Notably, PL at a much lower concentration (1.5 mg/kg) showed a comparable anticancer effect in HCC-bearing mice and increasing PL concentration did not significantly enhance its anticancer effects (Fig. 7A, 7B and 7C).

Consistent with *in vitro* assays, results of Western blotting analysis demonstrated that PL significantly activated p-PERK and up-regulated Ire 1α, PDI, and CHOP in H22 tumor xenografts (Fig. 7D). Meanwhile, MAPKs (i.e., p38, JNK and Erk) were significantly activated in H22 tumor xenografts (Fig. 7E). These results verified that the ER-MAPKs-CHOP signaling pathway was activated by PL in HCC cells *in vivo*.

## DISCUSSION

In the present study, we demonstrated that PL selectively killed HCC cells and inhibited their migration and invasion. PL activated ROS-ER-MAPKs-CHOP signaling axis prominently, which consequently suppressed HCC cell migration/invasion preferentially.

We demonstrated that PL was a selective anti-cancer drug for HCC. PL effectively induced cell death in various HCC cell lines including the highly malignant LM3 cells [33] with an IC_50_ of 10-20 μM but had little cytotoxicity in normal hepatic cells under similar conditions (Fig. 1). The cytotoxic efficacy of PL in HCC cells was similar to that in glioblastoma multiforme (GBM) cells [9], suggesting a general selective cytotoxicity of PL in those highly malignant cancers. In addition, PL selectively suppressed HCC migration/invasion but did not affect the migration of normal hepatic cells (Fig. 2). Notably, PL at a lower concentration (2.5-10 μM) exhibited a remarkable effect on suppressing HCC cell migration in scratched cultures in which PL-induced cell death was negligible (Fig. 2), suggesting a major anti-cancer effect of PL on suppressing metastasis. This is particularly important for the treatment of highly malignant tumors such as HCC since metastasis is the major cause of death [1-3]. Consistent with the anti-cancer effects of PL *in vitro* assays, PL was effective in reducing HCC development in mice (Fig. 7). PL might suppress HCC development *in vivo* via inducing cell death, suppressing cell migration/invasion and reducing tumor vessel formation (Supplemental Fig. 8). PL at 1.5-3.5 mg/kg/d showed comparable anticancer effects in HCC xenograft-bearing mice, suggesting that the therapeutic effects of PL *in vivo* reached a plateau at the dosage around 1.5 mg/kg/d and that further increasing the dosage of PL might not improve PL's therapeutic effect much due to the limited effective drug concentration in cancer tissues, the varied sensitivity of cancer cells to drug, the complicated feedback drug-resistant mechanisms or the increased side effects of drug *in vivo*. Since PL is a natural product which has been already used in traditional medicines for a thousand years [12, 13], it is practical to apply PL to the treatment of the highly malignant HCC clinically.

Our data clearly demonstrated that the ROS accumulation was a major initial factor responsible for PL's anticancer effects in HCC as pre-treatment of antioxidants completely abolished PL-induced cytotoxicity in HCC cells while delayed administration of antioxidants (3-12 h post PL treatment) markedly reduced their efficacy in antagonizing PL's cytotoxicity (Fig. 3E). Previous studies mainly focus on the cytotoxic effects of PL in cancer cells and various ROS-dependent cell death mechanisms, e.g., apoptosis [36-38], autophagic cell death [16, 39], cell cycle arrest [17, 38], GSTP1 [13], p38 [9, 39], JNK [9, 38], Erk [15], which have been proposed in different cancer cell lines. Until now, however, the exact molecular mechanisms of PL's action in cancer cells remain largely unclear, probably reflecting the nature of non-specific ROS stimulation in multiple signaling pathways and the complicated regulatory mechanisms involving in cell death process [5-9]. In HCC cells, the PL-induced cytotoxicity could not be efficiently reduced by a single signaling pathway inhibitor except antioxidants, suggesting that multiple ROS-dependent cell death mechanisms are involved in PL's cytotoxicity in HCC cells.

Unlike the elusive cell-death mechanisms underlying PL's action, we clarified a key signaling pathway (i.e., ROS-ER-MAPKs-CHOP) which was preferentially responsible for PL's action on HCC cell migration/invasion. Different from previous studies which showed a differentially activation of p38, JNK and Erk in distinct cancer cells [9,10,15], we demonstrated that PL induced prominent activation of all the three major MAPKs signaling pathways including p38, JNK and Erk in HCC cells, supporting that MAPKs were major ROS-stress responsive signaling pathways upon PL treatment. It is well-known that ER is a major organelle responsible for ROS accumulation [20] and MAPKs are canonical downstream signaling pathways of ER stress-responses [21]. Indeed, ROS-dependent activation of PERK, release of Ca^2+^ and induction of Ire 1α were prominent in HCC cells upon PL treatment (Fig. 4). Inhibition of ER stress-responses by 4-PBA suppressed PL-activated MAPKs (Fig. 6), supporting that PL activated the MAPKs via ER stress-responses in HCC cells. CHOP is a key ER stress-response product involved in cell death [21]. Interestingly, suppressing ER-MAPKs-CHOP signaling axis by either inhibitors (4-PBA, SP, SB, or U0126) or knocking-down of CHOP did not affect the rate of HCC cell death evidently but showed prominent effects on HCC cell migration, suggesting that PL preferentially suppressed HCC migration/invasion via ER-MAPKs-CHOP. Consistent with PL's effects on HCC cell migration, PL also suppresses cell migration of highly malignant GBM cells via ROS-p38/JNK/NFκB signaling [10]. These evidences suggest that MAPKs might be a signaling hub for PL's suppressed effects on cell migration/invasion. Further dissecting the molecular mechanisms underlying PL's effects on cancer cell migration/invasion is important for future applications of PL in cancer therapy.

In summary, we have demonstrated that PL selectively killed HCC cells and suppressed HCC cell migration/invasion. Preferentially, PL suppressed HCC migration/invasion via ROS-ER-MAPKs-CHOP axis signaling. Our data suggest that PL has the prospects of application in the treatment of highly malignant HCC.

## METHODS

### Cell culture, reagents and antibodies

Human HCC cell lines (HepG2, Huh7) and normal hepatic L-02 cells were purchased from ATCC (Manassas, VA, USA). HepG2 and Huh7 were cultured with 10% FBS in DMEM and L-02 was cultured with 10% FBS in RPMI-1640. Highly malignant human HCC LM3 cells were obtained from Zhongshan Hospital, Fudan University and cultured with 15% FBS in DMEM [40]. Mouse HCC cell line H22 was purchased from Shanghai SLAC Laboratory Animal Co. Ltd. (Shanghai, China). Primary cultures of rat hepatocytes were set as reported [41]. Briefly, hepatocytes (cell viability >85%) were isolated from livers of normal male Wistar rats by collagenase perfusion and mechanical disruption, seeded in collagen-coated 96-well plates with 10% FBS in high-glucose DMEM at a density of 2×10^4^ cells/cm^2^, and used at day 5 *in vitro*. All culture media and fetal bovine serum (FBS) were purchased from Gibco/Life Technologies (Carlsbad, CA, USA). Piperlongumine, 5-fluorouracil (5-FU), 4-phenylbutyric acid, Hoechst 33342, propidium iodide, Fluo-3 AM, trypan blue, glutathione, N-acetyl-L-cysteine (NAC), 2,7-dichlorodihydrofluorescein diacetate (DCFH-DA), 3-(4,5-dimethylthiazol-2-yl)-2,5-diphenyltetrazolium bromide (MTT), 3-Methyladenine (3-MA) and crystal violet were purchased from Sigma-Aldrich (St Louis, MO, USA). SB203580 (p38 pathway inhibitor), SP600125 (JNK pathway inhibitor), U0126 or PD98059 (MEK 1/2 inhibitors), LY294002 (PI-3K inhibitor) and Go6983 (PKCζ inhibitor) were purchased from Cell Signaling Technology (Danvers, MA, USA). Annexin V-FITC apoptosis detection kit was purchased from KeyGEN BioTECH (Nanjing, China). Quantification of reduced GSH was conducted by a kit from Nanjing Jiancheng Bioengineering Institute (Nanjing, China). Antibodies against β-actin were purchased from Santa Cruz Biotechnology (Santa Cruz, CA, USA), and antibodies against Grp78, PDI, p-PERK (Thr981)/PERK, Ire 1α, CHOP, p-p38 (Thr180/Tyr182)/p38, p-JNK (Thr183/Tyr185)/JNK, p-Erk (Thr202/Tyr204)/Erk were from Cell Signaling Technology (Danvers, MA, USA).

### Piperlongumine and other drug treatments

PL was dissolved in dimethyl sulfoxide (DMSO) at a stock concentration of 50 mM and used at designated final concentration (0-20 μM) with a maximal DMSO concentration less than 0.5% DMSO. DMSO of the same concentration in DMEM was used as the vehicle control (i.e., PL 0). NAC, GSH and SB203580/SP600125/U0126 were used at 3 mM, 5 mM and 10 μM respectively. 4-PBA was used at a final concentration ranged from 1-5 mM. NAC and other drugs were added simultaneously with PL at designated final concentrations or as indicated.

### Cell viability or cell death assays

Cell viability was determined by MTT assay and cell death was determined by trypan blue exclusion assay or PI staining. For MTT assays, 5000 cells/well were seeded in 96-well plates. After 24 h of incubation, cells were treated with drugs in six parallel wells for designated times and then assayed with MTT as reported [42, 43]. For trypan blue exclusion assay and PI staining, cells at 60–70% confluence in six-well plates were treated with drugs in triplicates for 24 h and then subjected for corresponding assays.

### Flow cytometry assay

FCM assay was employed to analyze apoptosis and cell cycle as previously reported [9]. HCC cells were treated with PL for 24 h and co-stained with annexin V-FITC and PI according to the manufacturer's instructions (KeyGEN BioTECH). Apoptotic cells were separated and quantified by a FACSCalibur Flow Cytometry System (Becton Dickinson, San Jose, CA, USA). For cell cycle assay, HCC cells were treated with PL for 24 h and stained with PI [10].

### Scratch-wound healing assay

Cell migration after scratching was performed as reported previously [10]. Briefly, confluent cells in 35-mm dishes were scratched with 200-μl pipette tips. Immediately after cell scratching, the wounded edges were micrographed (designated as 0 h). Scratched cells were then incubated with fresh complete media with appropriate concentration of drugs and the wounded edges were micrographed at designated time points. The wound size (i.e., the gap between the two opposite wounded edges) was calculated using the Image-Pro Plus software (Media Cybernetics, USA) and the average of wound size represented at least 12 different fields in each culture dish. Relative wound sizes of designed time points after scratching were compared to that of 0 h control.

### Transwell migration and invasion assays

Transwell migration and invasion assays were performed as reported [10, 44]. Briefly, HepG2 cells (1×10^5^ cells/well) were seeded into the upper chamber of a transwell apparatus with a 8-μm pore size membrane (Corning Incorporated, Corning, NY, USA) in 200 μl of serum-free DMEM containing drugs. For the invasion assay, the upper chamber was pre-coated with matrigel basement membrane matrix (BD Bioscience, Bedford, MA, USA) according to the manufacturer's protocols. For both assays, the medium of lower chamber contained 800 μl of complete medium. After 24 h, the migrated or invaded cells at the lower surface of the filter were stained, micrographed and calculated as reported previously [10]. The average migrated/invaded cell number represented at least nine different fields in each filter.

### Measurement of intracellular ROS and reduced glutathione (GSH)

Intracellular ROS level was measured using DCFH-DA as reported previously [9, 10]. Briefly, PL-treated cells were incubated with 10 μM of DCFH-DA for 30 min at 37°C, washed twice with PBS and then micrographed with a conventional fluorescent microscope (Olympus, Tokyo, Japan). For each culture, a minimum of 9 random fields were captured. Average fluorescent intensity was analyzed using the Image-Pro Plus software (Media Cybernetics, USA). For GSH measurement, PL-treated HepG2 or Huh7 cells were washed with ice-cold PBS three times, scrapped off from the plates and subjected to sonification. The supernatants were collected and reduced GSH was calculated according to the manufacturer's instructions (Nanjing Jiancheng Bioengineering Institute).

### Western blotting analysis and fluorescent cytoimmunostaining

Western blot analysis and fluorescent cytoimmunostaining were performed as described previously [45]. Briefly, equal amounts of total proteins were subjected to mini-PAGE gel electrophoresis and transferred to NC membranes. The blots were incubated with corresponding primary and fluorescent secondary antibodies. The bands were quantified using the Odyssey Infrared Imaging System (LI-COR Bioscience, Lincoln, NE, USA). β-actin was used as internal control. For fluorescent cytoimmunostaining, PL-treated cells in the 35-mm culture dishes were fixed, permeabilized, blocked, and then incubated with corresponding primary and Dylight 488-labeled secondary antibodies (Abbkine, Redlands, CA, USA). Micrographs were taken under the same conditions with a conventional fluorescent microscope (Olympus, Tokyo, Japan).

### Intracellular Ca^2+^ measurements

Intracellular Ca^2+^ levels were detected with Fluo-3 AM as reported [46]. PL-treated cells were washed with Ca^2+^-free PBS three times and then incubated with 5 μM of Fluo-3AM for 30 min. After brief rinse with Ca^2+^-free PBS, living cells were micrographed immediately under a conventional fluorescent microscope (Olympus, Tokyo, Japan).

### RNA interference

Small interfering RNA for CHOP (5′-GGUAUGAGGACCUGCAAGA-3′, si-CHOP) was chemically synthesized (Guangzhou RiboBio Co., Ltd., Guangzhou, China) and non-specific control si-GFP was provided by Guangzhou RiboBio. Twenty-four hours after cell seeding, interfering RNAs at 50 nM were transfected into HepG2 cells using Lipofectamine 2000 (Invitrogen, Carlsbad, CA, USA) as previously reported [45]. Two days after siRNA trensfection, the cultures were subjected to PL treatment and the corresponding assays.

### Hepatocarcinoma 22 (H22)-derived solid HCC in mice

Specific pathogen-free male Kunming mice with body weights of 18-22g were purchased from the Experimental Animal Centre, Huazhong University of Science and Technology (HUST). All the mice were housed in a clean environment and had free access to standard rodent diet and water at room temperature and a humidity of 45–55%. All the animal experimental procedures are approved by the Animal Care and Use Committee of Tongji Medical College, HUST. H22-derived solid HCC in mice was generated by injecting 2×10^7^ H22 cells (in 0.2 ml of PBS) into the left armpits of mice as described [47]. Twenty-four hours after initial H22 inoculation, 50 mice were randomly divided into five groups (i.e., DMSO, PL 1.5 mg/kg.day, PL 2.5 mg/kg.day, PL 3.5 mg/kg.day and 5-FU 20 mg/kg.day; 10 mice/group) and PL or 5-Fu was delivered as designated by i.p. once per day for 14 days consecutively. H22-derived tumor xenografts were harvested, measured and weighted on the 15^th^ day of H22 inoculation as described previously [43].

### Statistical analyses

All experiments were repeated independently at least three times. The values were expressed as mean±SEM and statistics were performed with a 2-way ANOVA followed by the Student-Newman-Keuls test. *P* values of less than 0.05 were considered statistically significant.

## SUPPLEMENTARY MATERIAL FIGURES



## Abbreviations

4-PBA: 4-phenylbutyric acid
5-FU: 5-fluorouracil
CHOP: C/EBP homologous protein
DCFH-DA: 2′-,7′-Dichlorofluorescin diacetate
3-MA: 3-Methlylaclenine
DMEM: Dulbecco's modified Eagle's medium
DMSO: dimethyl sulfoxide
ER: endoplasmic reticulum
Erk: extracellular signal-regulated kinase
FBS: fetal bovine serum
FCM: flow cytometry
GFP: green fluorescent protein
Grp78: glucose-regulated protein 78
GSH: glutathione
HCC: hepatocellular carcinoma
JNK: c-Jun N-terminal kinase
IC_50_: half maximal (50%) inhibitory concentration
i.p.: intraperitoneal
Ire 1α: inositol-requiring enzyme 1 alpha
MAPKs: mitogen-activated protein kinases
MTT: 3-(4,5-Dimethylthiazol-2-yl)-2,5-diphenyltetrazolium bromide
NAC: N-acetyl-L-cysteine
NFκB: nuclear factor kappa B
PBS: phosphate buffered saline
PDI: protein disulfide isomerase
PERK: PKR-like ER kinase
PI: propidium iodide
PL: piperlongumine
ROS: reactive oxygen species
siRNA: small interfering RNA.
