# Supplementary material for: Piperlongumine selectively kills hepatocellular carcinoma cells and preferentially inhibits their invasion via ROS-ER-MAPKs-CHOP
Source: Oncotarget. 2015 Jan 31;6(8):6406–21. doi: 10.18632/oncotarget.3444 (PMC4467445; doi:10.18632/oncotarget.3444)
Supplement: Supplementary file 1 [file oncotarget-06-6406-s001.pdf]

Piperlongumine selectively kills hepatocellular carcinoma cells and preferentially inhibits their invasion via ROS-ER-MAPKs-CHOP

Supplemental materials and methods

Tube formation assay

Human umbilical endothelial cells (HUVEC) were obtained from freshly isolated umbilical veins as described previously [1]. Briefly, the umbilical vein was rinsed with PBS and digested with 0.25% trypsin for 30 min sterilely. The collected HUVEC were then cultured with endothelial cell medium (ECM) complete media containing endothelial cell growth supplement (ECGS) and 5% FBS (ScienCell, Carlsbad, USA) and were used within 9 passages for the tube formation assay. The tube formation assay was performed as reported [2-4]. Briefly, HepG2 cells were seeded onto 24-well plates in DMEM containing 10% FBS at a density of 10×10^4 cells/well. One day after seeding, HepG2 cells were treated with piperlongumine (0, 1, 2, or 5 μM) in DMEM containing 2% FBS for 24 h and the conditioned media were collected following centrifugation. Matrigel matrix (Basement membrane matrix, BD Bioscience, Bedford, MA, USA) was thawed at 4°C overnight, added onto the 96-well plates (50 μl/well) and gelled at 37°C for 1 h. Then, HUVEC cells at a density of 8×10^4 cells/well in 200 μl of conditioned media were overlaid onto the matrix gels. After incubation for 8 h, HUVEC tubes were microphotographed using a conventional microscope. Tube formation parameters (i.e., tube length, mesh area, the number of meshes and the number of branching point) were analyzed using the Image-Pro Plus image analysis software. The data represented the mean values of at least six fields per well as compared to PL 0.

Supplemental Figures

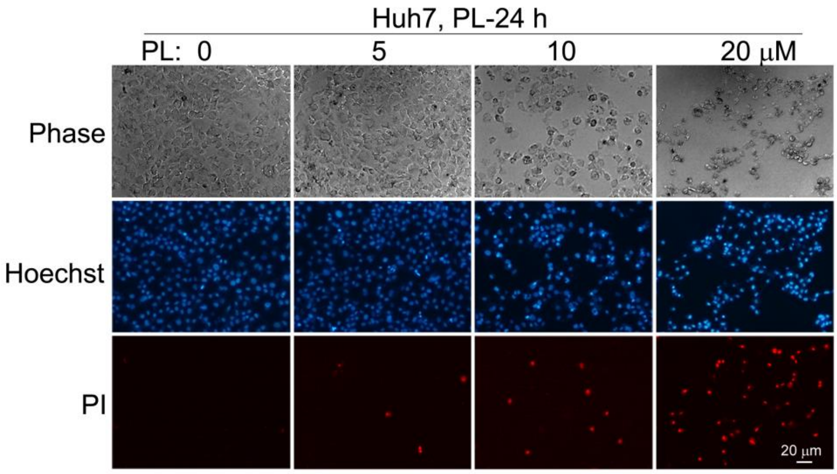

Figure S1: Representative micrographs PI/Hoechst staining showing the effects of PL treatment on cell death in Huh7 cells. Huh7 cells in 24-well plates at 24 h after seeding were incubated with various concentrations (0, 5, 10 or 20 μM) of PL for 24 h. The cultures were co-stained with PI and Hoechst 33342 before microscopy.

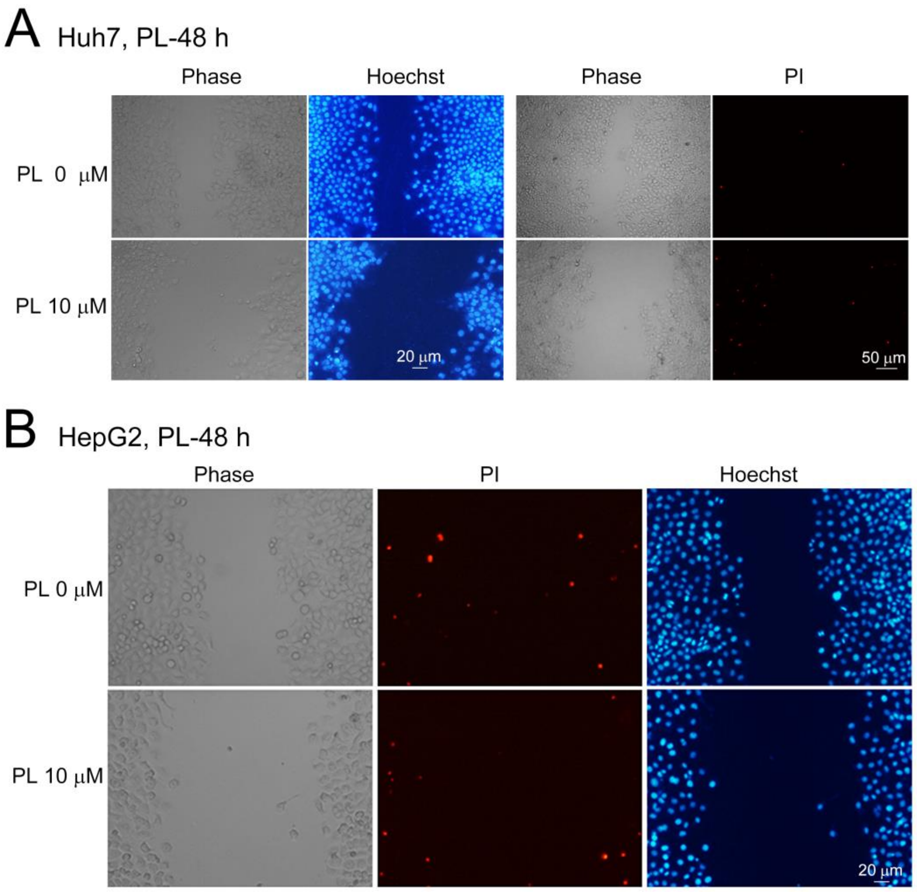

Figure S2: Representative micrographs of PI/Hoechst staining showing the effects of PL treatment on cell death after cell starching in Huh7 (A) and HepG2 (B) cells. Confluent Huh7 (A) and HepG2 (B) were subjected to cell scratching and PL treatment simultaneously. PI and/or Hoechst staining were performed at 48 h after cell scratching. Representative results showed that PL at 10 μM did not affect cell death in Huh7 (A) and HepG2 (B) cells at 48 h after cell scratching.

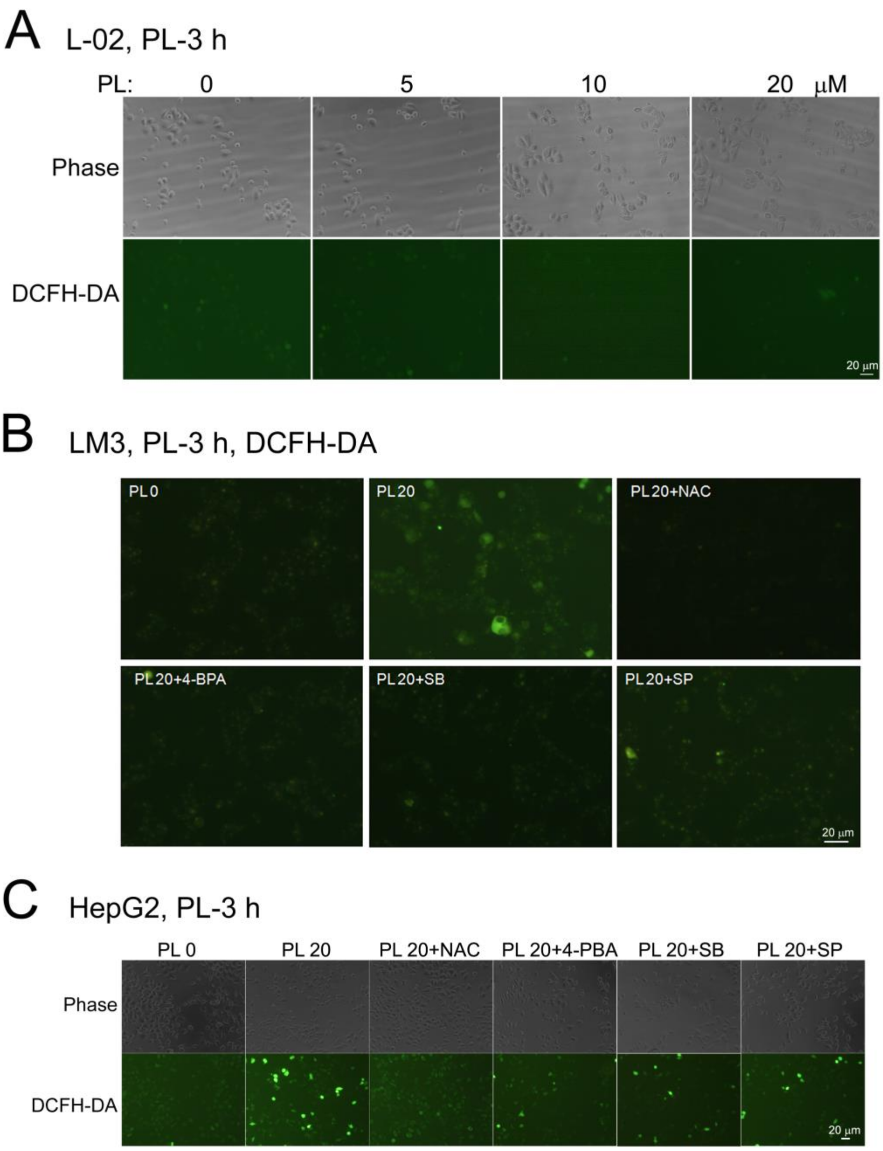

Figure S3: Representative micrographs of DCFH-DA staining showing the effects of PL and other drugs on ROS levels in L-02 (A), LM3 (B) and HepG2 (C) cells. PL was administrated alone (0, 5, 10 or 20 μM) or together with other drugs (i.e., NAC 3 mM, 4-PBA 5 mM, SB203580 10 μM or SP600125 10 μM) for 3 h before DCFH-DA staining.

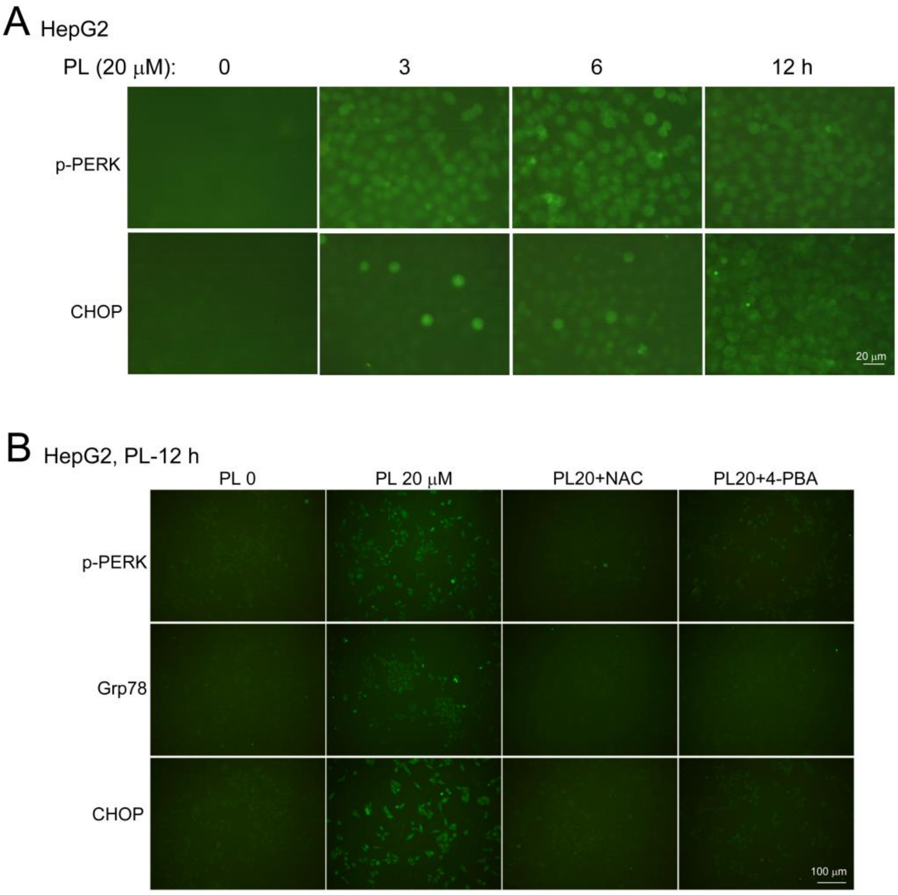

Figure S4: Effects of PL, NAC or 4-PBA on p-PERK, CHOP and Grp78 expression in HepG2 cells. (A) Representative results of fluorescent cytoimmunostaining showed that p-PERK and CHOP were up-regulated in HepG2 cells upon 20 μM of PL treatment at various time points. (B) Representative results of fluorescent cytoimmunostaining showed that co-treatment of NAC (3mM) or 4-PBA (5mM) with PL (20 μM) effectively abolished PL-elevated p-PERK, Grp78 or CHOP in HepG2 cells.

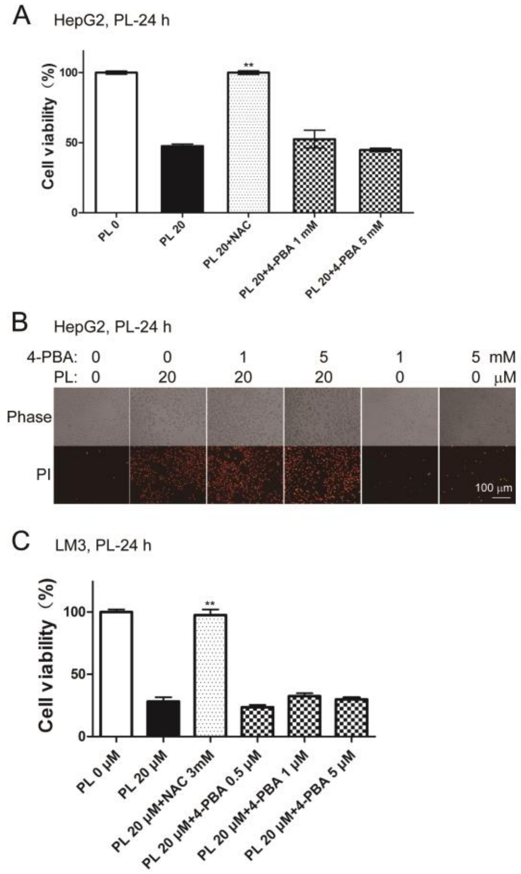

Figure S5: Effects of 4-PBA on PL-induced cell death in HCC cells. (A) Effect of 4-PBA on cell viability (MTT assay) in HepG2 cells. n=3. All values represented mean ± SEM of three independent experiments. ***P*<0.01 *vs.* corresponding PL-20 μM. (B) Representative micrographs of PI staining showed the effect of 4-PBA on PL-induced cell death in HepG2 cells. (C) Effect of 4-PBA on cell viability (MTT assay) in LM3 cells. n=3. **P*<0.05 *vs.* corresponding PL-20 μM.

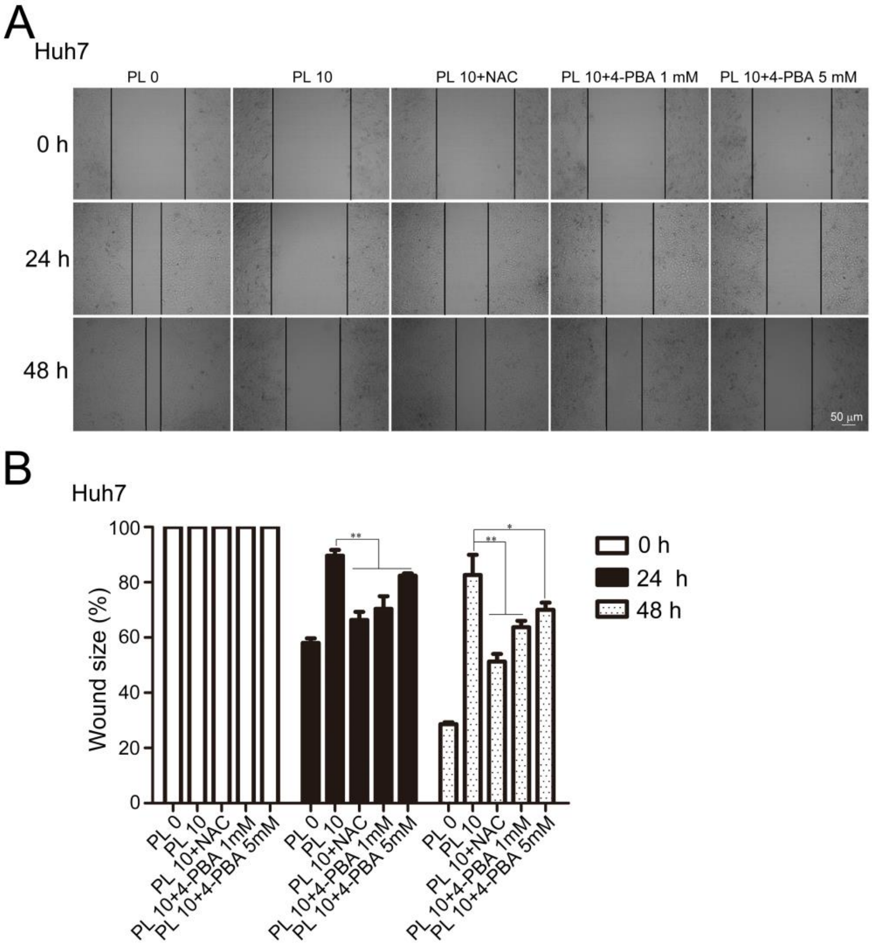

Figure S6: Effect of NAC or 4-PBA on PL-suppressed Huh7 cell migration after scratching. (A) Representative micrographs showed the effect of co-incubation of NAC (3 mM) or 4-PBA (1 or 5 mM) with PL on Huh7 cell migration at various time points after scratching. (B) Results of statistical analysis demonstrated that NAC or 4-PBA significantly reversed PL-suppressed Huh7 cell migration at 24 or 48 h after cell scratching. * *P*<0.05, ***P*<0.01 *vs* PL 10 (n=3).

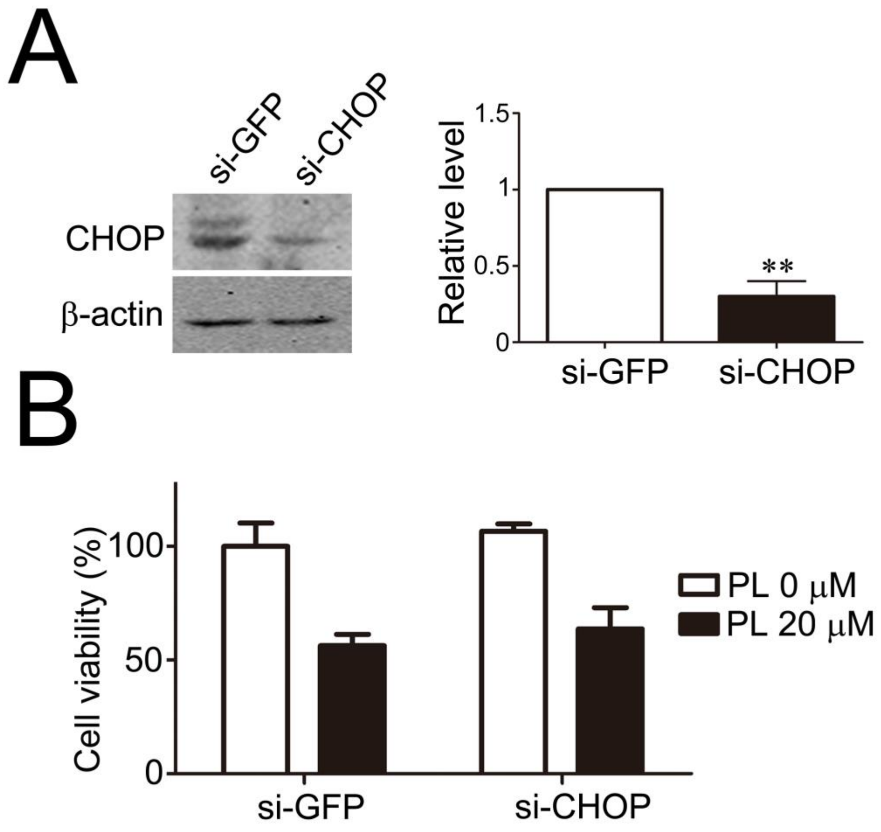

Figure S7: Effect of CHOP knock-down on cell viability in HepG2 cells. (A) Results of Western blotting analysis demonstrated that si-CHOP significantly reduced CHOP expression in HepG2 cells. HepG2 cells were transfected with small interfering RNA specific to CHOP (si-CHOP, 50 μM) or non-specific si-GFP control for 48 h and subjected to Western blotting analysis. $^{**}P<0.01$ *vs* si-GFP (n=3). (B) Effect of CHOP knock-down on the cell viability (MTT assay) in HepG2 cells. HepG2 cells were transfected with si-CHOP or si-GFP for 48 h and subjected to MTT assay.

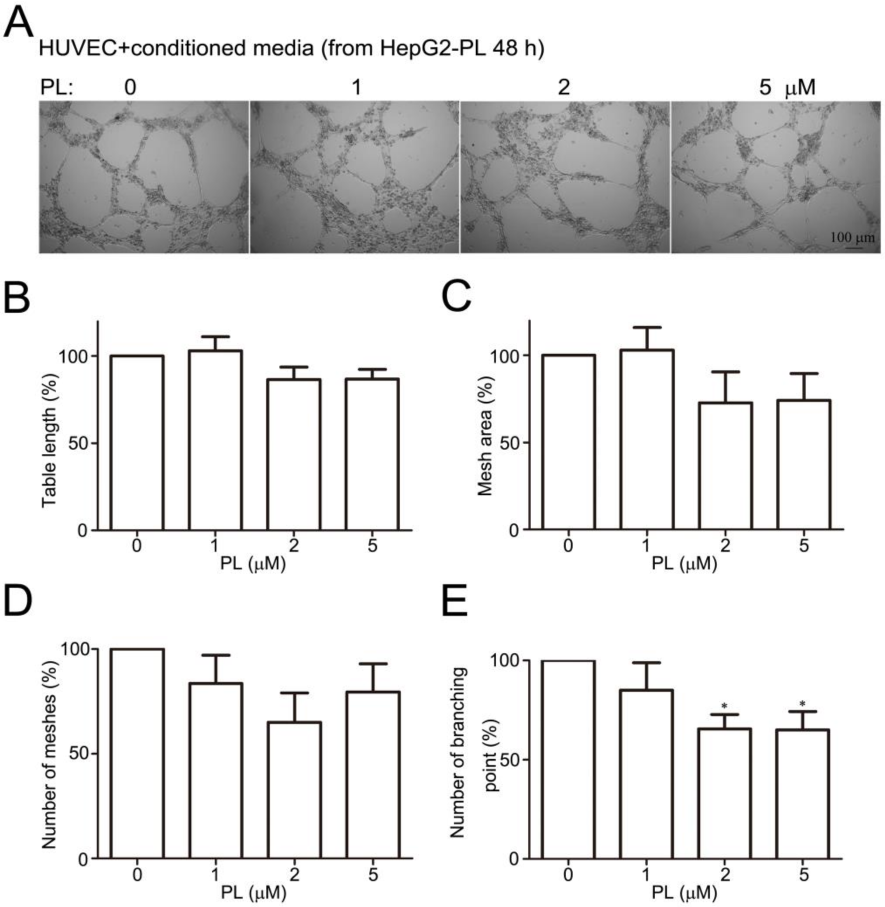

Figure S8: Effects of PL-treated HepG2 cells on HUVEC tube formation. (A) Representative micrographs showed the effect of PL-treated HepG2 cells on HUVEC tube formation. HepG2 cells were treated with various concentrations of PL for 48 h and the conditioned media were collected. HUVEC on the Matrigel matrix were incubated with the conditioned media from PL-treated HepG2 cells for 8 h and micrographs were taken under lower magnifications. (B)-(E) Statistical analysis of the tube length (B), the mesh area (C), the number of meshes (D) and the number of branching point (E) of HUVEC upon conditioned-media incubation obtained from PL-treated HepG2 cells. *$P<0.05$ *vs* PL 0 (n=3).

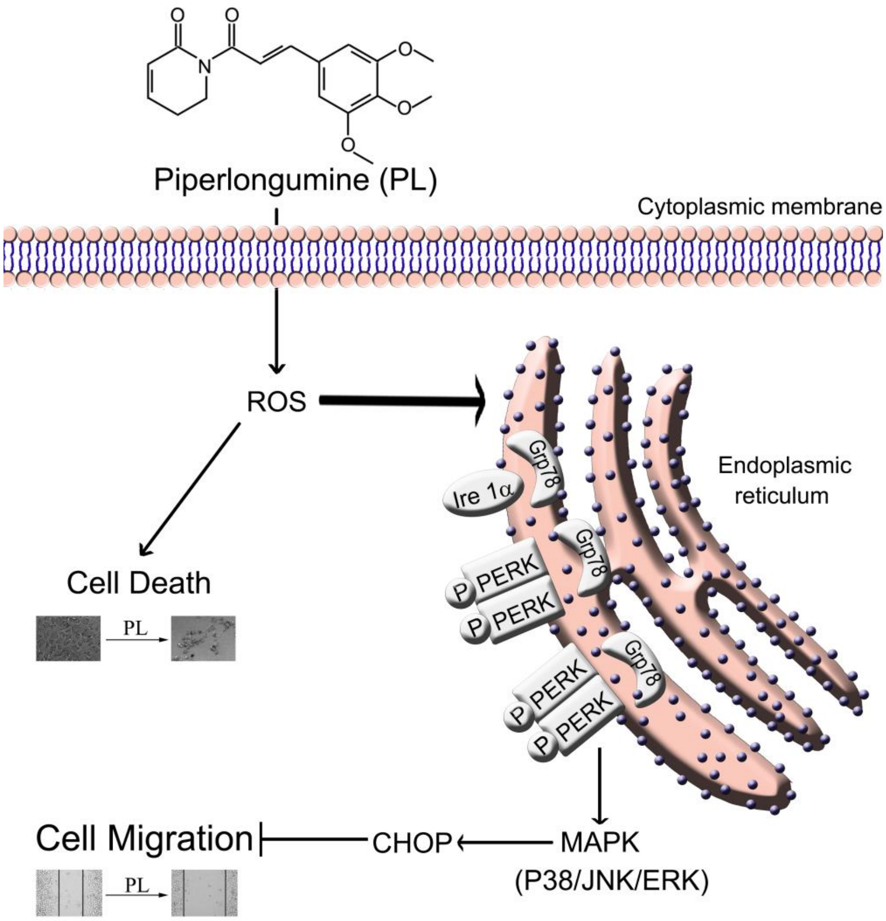

Figure S9: Proposed mechanisms of PL's action on HCC cell migration. PL induces ROS accumulation which subsequently induces ER stress-responses including the activation of PERK and induction of Grp78/Ire 1α. Following ER stress-responses, cytoplasmic MAPKs and CHOP are activated or up-regulated, which finally contribute specifically to PL-suppressed cell migration/invasion in HCC cells. ⟶, induction or activation; ⊥, inhibition.

Supplemental References

1. Bian F, Yang X, Zhou F, Wu PH, Xing S, Xu G, Li W, Chi J, Ouyang C, Zhang Y, Xiong B, Li Y, Zheng T, Wu D, Chen X and Jin S. C-reactive protein promotes atherosclerosis by increasing LDL transcytosis across endothelial cells. Br J Pharmacol. 2014; 171(10):2671-2684.
2. Porcu E, Viola G, Bortolozzi R, Persano L, Mitola S, Ronca R, Presta M, Romagnoli R, Baraldi PG and Basso G. TR-644 a novel potent tubulin binding agent induces impairment of endothelial cells function and inhibits angiogenesis. Angiogenesis. 2013; 16(3):647-662.
3. Guidolin D, Vacca A, Nussdorfer GG and Ribatti D. A new image analysis method based on topological and fractal parameters to evaluate the angiostatic activity of docetaxel by using the Matrigel assay in vitro. Microvasc Res. 2004; 67(2):117-124.
4. Hu L, Duan YT, Li JF, Su LP, Yan M, Zhu ZG, Liu BY and Yang QM. Biglycan enhances gastric cancer invasion by activating FAK signaling pathway. Oncotarget. 2014; 5(7):1885-1896.
